# Jcvrisk: An R Package for Population-level Estimation of Cardiovascular Risk Scores in Japanese Adults

**DOI:** 10.2188/jea.JE20250292

**Published:** 2026-04-05

**Authors:** Hiroshi Okumiyama, Ryosuke Fujii, Yoshiki Tsuboi, Kazuma Murakami, Riku Umematsu, Yoshitaka Ando, Hiroaki Ishikawa, Genki Mizuno, Koji Ohashi, Hiroya Yamada, Mirai Yamazaki, Koji Suzuki

**Affiliations:** 1 Department of Preventive Medical Sciences, Fujita Health University School of Medical Sciences, Aichi, Japan; 2 Department of Informative Clinical Medicine, Fujita Health University School of Medical Sciences, Aichi, Japan; 3 Department of Medical Technology, Tokyo University of Technology School of Health Sciences, Tokyo, Japan; 4 Department of Hygiene, Fujita Health University School of Medicine, Aichi, Japan

**Keywords:** cardiovascular disease, coronary heart disease, ischemic stroke, risk score, epidemiology

## Abstract

**Background:**

Cardiovascular disease (CVD) remains a leading cause of death in Japan. Although several CVD risk scores tailored for Japanese individuals have been developed, no tools are available to estimate these scores at the population level. We developed the “Jcvrisk” R package, which integrates four major Japanese CVD risk models recommended by the clinical guideline from the Japanese Circulation Society (JCS). As a showcase, we applied the Jcvrisk package to longitudinal population-based study to evaluate trends in estimated different risk scores.

**Methods:**

We used longitudinal data from the Yakumo Study, an annual health checkup for residents in Yakumo, Hokkaido. This package includes four risk models with 14 risk scores from representative cardiovascular cohort studies, including three Evidence for Cardiovascular Prevention From Observational Cohorts scores, one Hisayama score, two Suita scores, and eight Japan Atherosclerosis Longitudinal Study scores. For temporal comparisons of CVD risk scores, we summarized scores from 2000 to 2020 every 5 years.

**Results:**

The mean age of participants throughout all study years was around 60 years. Most risk factors did not change remarkably over the 20 years, with only a decrease in smoking prevalence and an increase in high density lipoprotein cholesterol. However, all CVD risk scores consistently indicated an upward trend in 10-year CVD risk.

**Conclusion:**

Jcvrisk package includes functions to calculate CVD risk scores for Japanese adults. The package serves as a valuable tool for researchers and policymakers aiming to assess and monitor cardiovascular risk at both individual and the population level in Japan.

## INTRODUCTION

Cardiovascular disease (CVD) is a major global health concern. In Japan, CVD and cerebrovascular disease together accounted for 335,681 deaths (21.3% of all-cause mortality) in 2023, ranking second only to cancer, which caused 382,504 deaths (24.3%).^1^ Given this appalling situation, identifying an at-risk population is the first step in preventing CVD.

To quantify individual risk, CVD risk scores estimate disease onset and mortality using demographic, clinical, and lifestyle factors. In the United States, starting from the Framingham Risk Score (FRS),^2^ the American college of cardiology and the American Heart Association introduced the Pooled Cohort Equation,^3^ and recently announced PREVENT.^4^ In European countries, at the same time, the European Society of Cardiology recommends the use of SCORE2.^5^ However, these models often perform poorly in other ethnic groups.^6^ In Japan, several CVD risk scores have been developed.^7^^–^^10^ Of these, four scores from large cohort studies are featured in the 2023 “Guidelines for the Primary Prevention of Coronary Artery Disease” by the Japanese Circulation Society (JCS).^11^ Each score differs in target outcomes, predictors, and timeframes.

While tools existed for individual risk estimation, a new calculator is expected to simultaneously calculate all risk scores, which may contribute to policy making of cardiovascular disease at the population level (eg, large health insurance associations and local governments). In addition, the integrative tool would enhance for cardio-epidemiological research in Japan. Therefore, we aimed to develop an R (R Foundation for Statistical Computing, Vienna, Austria) package “Jcvrisk” which can integrate different CVD risk scores for Japanese adults using tabular data. We applied it to longitudinal dataset to demonstrate population-level risk transitions over 20 years.

## METHODS

### Jcvrisk package

**Figure 1.**
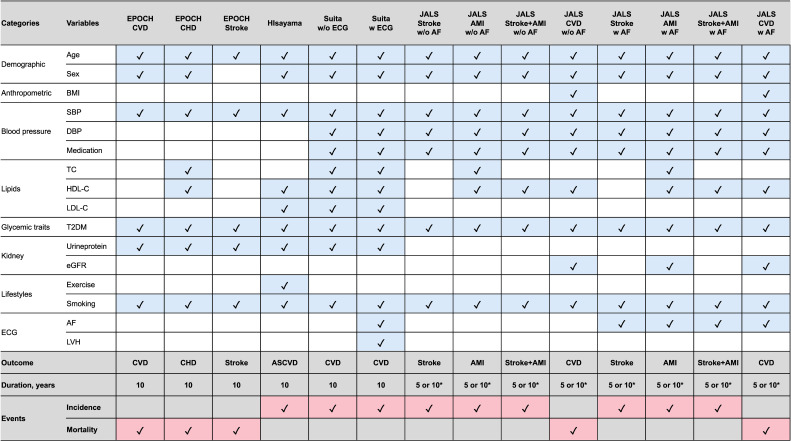
Key variables and target outcomes across 14 different cardiovascular disease risk score for Japanese adults. ^*^The original paper for the JALS scores provided absolute risks both for 5 and 10 years. However, in accordance with the authors’ intentions, the Jcvrisk package implements the absolute risk over 5 years. AF, arterial fibrillation; AMI, acute myocardial infarction; ASCVD, atherosclerotic cardiovascular disease; BMI, body mass index; CHD, coronary heart disease; CVD, cardiovascular Disease; DBP, diastolic blood pressure; ECG, electrocardiogram; eGFR, estimated glomerular filtration rate; EPOCH, Evidence for Cardiovascular Prevention From Observational Cohorts; HDL-C, high-density lipoprotein cholesterol; JALS: Japan Atherosclerosis Longitudinal Study; LDL-C, low-density lipoprotein cholesterol; LVH, left ventricular hypertrophy; SBP, systolic blood pressure; TC, total cholesterol; T2DM, Type 2 diabetes.

The “Jcvrisk” R package calculates 14 CVD risk scores introduced in the JCS guideline: 1) Evidence for Cardiovascular Prevention From Observational Cohorts (EPOCH) for CVD,^7^ 2) EPOCH for coronary heart disease (CHD),^7^ 3) EPOCH for stroke,^7^ 4) Hisayama for atherosclerotic cardiovascular disease (ASCVD),^8^ 5) Suita with electrocardiogram (ECG),^9^ and 6) Suita without ECG,^9^ 7) Japan Atherosclerosis Longitudinal Study (JALS) for stroke, 8) JALS for acute myocardial infarction (AMI), 9) JALS for stroke + AMI, 10) JALS for CVD without atrial fibrillation (AF),^10^ and 11–14) 7–10) with AF^10^ (Figure 1). All functions take as input a data frame with required variable names. Column order does not matter, but specified names must match. For details, please refer to the official documentation (https://cran.r-project.org/web/packages/Jcvrisk/index.html) and eMaterial 1. Warnings appear if data fall outside reference ranges or contain missing values; in such cases, complete case analysis is used.

### Target populations

As a showcase, we used longitudinal data from the Yakumo Study. This study is based on a health checkup in Yakumo, Hokkaido, and has been annually conducted since 1982. The eligible participants are residents aged 40 years or older who participate in the health checkup. In addition to regular health checkup items, Yakumo Study also collects several items used for research, such as ultrasound examinations and cognitive function tests. The participants were mainly aged from late 50s to early 60s, with women accounting for about 60%. We analyzed data from 2000 to 2020 every 5 years. Due to the coronavirus disease 2019 pandemic, data for 2020 was not available and data for 2019 were substituted. Additional information is summarized in previous papers.^12^^,^^13^ Written informed consent was obtained from all participants before the study began. The study protocol was approved by the Ethical Review Committee of Fujita Health University (HM: 24-578).

### Data collection

Information on age, sex, exercise habits, smoking status, fasting time, and antihypertensive use was collected using a self-administered questionnaire. Blood pressure (systolic blood pressure [SBP] and diastolic blood pressure [DBP]) was both measured at a resting state at a health checkup site. Proteinuria was determined using the urine dipstick test. Type 2 diabetes (T2DM) was defined as having HbA1c of 6.5% or higher, fasting blood glucose (fasting for 10 hours or longer) of 100 mg/dL or higher, or taking medications for T2DM. AF and left ventricular hypertrophy were defined using Minnesota codes which a physician classified based on electrocardiograms. Serum creatinine, total cholesterol, high-density lipoprotein cholesterol (HDL-C), and low-density lipoprotein cholesterol (LDL-C) were measured using the autoanalyzer at Yakumo General Hospital. Estimated glomerular filtration rate (eGFR) was calculated using the following formula^14^: 194 × serum creatinine^−1.039^ × age^−0.287^ × (0.739 if female).

### Statistical analysis

Estimated 10-year CVD risks were summarized using median and interquartile ranges. Of CVD risk scores, two Suita scores with and without ECG were classified into six risk categories (<1%, 2%, 4%, 6%, 9%, 14% or 15%, and >25% or >26%). All statistical analysis was performed by R software (ver. 4.3.2).

## RESULTS

The mean age of participants throughout all study years was around 60 years, and the proportion of women was slightly higher than that of men (eTable 1). Mean values of SBP and DBP were around 126–138 mm Hg and 76–80 mm Hg, respectively. HDL-C levels increased with each study year. The prevalence of T2DM was less than 10% for all study years, and the percentage of proteinuria varied slightly from 4 to 15%. The percentage of current smokers decreased from 24% to 15% in the past 20 years. Those who exercised habitually accounted for one-fifth to one-third of all respondents.

### EPOCH scores

All EPOCH scores reveal a consistent increase in the 10-year risk of CVD, CHD, and stroke incidence from 2000 to 2019 (Table 1). For CVD, the 10-year risk has risen steadily from 0.82% in 2000 to 1.66% in 2019. This trend reflects a gradual but notable escalation in cardiovascular risk over the two decades. Similarly, the probability of CHD events increased from 1.09% in 2000 to 2.16% in 2019, suggesting a growing likelihood of coronary events. For stroke, the 10-year probability shows a smaller yet consistent increase, starting at 0.59% in 2000 and reaching 1.19% in 2019.

**Table 1.** 10-year absolute risks estimated by 14 different cardiovascular disease risk scores from 2000 to 2019

CVD risk scores		2000	2005	2010	2015	2019
EPOCH CVD^a^		0.82 [0.19–2.11]	0.94 [0.30–2.38]	1.34 [0.42–3.26]	1.65 [0.59–3.43]	1.66 [0.60–3.48]
EPOCH CHD^a^		1.09 [0.21–3.14]	1.33 [0.38–3.42]	1.62 [0.48–4.55]	2.38 [0.73–5.06]	2.16 [0.78–5.20]
EPOCH Stroke^a^		0.59 [0.13–1.44]	0.65 [0.21–1.60]	0.85 [0.30–2.22]	1.11 [0.36–2.24]	1.19 [0.41–2.22]
Hisayama^a^		2.56 [1.45–5.98]	2.56 [1.26–5.98]	3.41 [1.67–7.89]	4.52 [2.22–9.06]	3.92 [1.93–7.89]
Suita^b^	<1%	11 (2.5%)	6 (1.7%)	4 (1.2%)	3 (0.9%)	6 (1.9%)
	2%	231 (52.9%)	172 (50.1%)	151 (45.6%)	126 (38.3%)	126 (40.0%)
	6%	70 (16.0%)	60 (17.5%)	55 (16.6%)	66 (20.1%)	59 (18.7%)
	9%	67 (15.3%)	56 (16.3%)	52 (15.7%)	63 (19.1%)	56 (17.8%)
	14%	35 (8.0%)	39 (11.4%)	50 (15.1%)	49 (14.9%)	40 (12.7%)
	25%	23 (5.3%)	10 (2.9%)	19 (5.7%)	22 (6.7%)	28 (8.9%)
Suita with ECG^b^	<1%	11 (2.5%)	6 (1.7%)	4 (1.2%)	3 (0.9%)	6 (1.9%)
	2%	230 (52.6%)	172 (50.1%)	151 (45.6%)	126 (38.3%)	126 (40.0%)
	6%	76 (17.4%)	61 (17.8%)	60 (18.1%)	70 (21.3%)	60 (19.0%)
	9%	63 (14.4%)	63 (18.4%)	54 (16.3%)	70 (21.3%)	62 (19.7%)
	15%	38 (8.7%)	31 (9.0%)	41 (12.4%)	39 (11.9%)	41 (13.0%)
	26%	19 (4.3%)	10 (2.9%)	21 (6.3%)	21 (6.4%)	20 (6.3%)
JALS Stroke without AF^a^		0.44 [0.25–0.93]	0.50 [0.27–0.98]	0.62 [0.33–1.11]	0.66 [0.38–1.07]	0.71 [0.38–1.28]
JALS AMI without AF^a^		0.12 [0.06–0.29]	0.10 [0.06–0.24]	0.13 [0.06–0.28]	0.15 [0.06–0.38]	0.12 [0.05–0.29]
JALS Stroke + AMI without AF^a^		0.54 [0.32–1.08]	0.63 [0.32–1.08]	0.72 [0.42–1.43]	0.82 [0.47–1.33]	0.82 [0.45–1.53]
JALS CVD without AF^a^		0.25 [0.14–0.50]	0.26 [0.13–0.44]	0.31 [0.17–0.92]	0.35 [0.20–0.80]	0.32 [0.20–0.86]
JALS Stroke with AF^a^		0.44 [0.26–0.93]	0.51 [0.27–0.93]	0.58 [0.31–1.15]	0.63 [0.36–1.07]	0.71 [0.39–1.20]
JALS AMI with AF^a^		0.11 [0.05–0.27]	0.11 [0.06–0.23]	0.14 [0.06–0.28]	0.15 [0.06–0.38]	0.12 [0.05–0.29]
JALS Stroke + AMI with AF^a^		0.54 [0.32–1.08]	0.64 [0.32–1.11]	0.73 [0.41–1.41]	0.82 [0.45–1.31]	0.82 [0.47–1.51]
JALS AMI without AF^a^		0.25 [0.14–0.50]	0.26 [0.13–0.44]	0.31 [0.17–0.92]	0.35 [0.20–0.80]	0.32 [0.20–0.86]

AF, arterial fibrillation; AMI, acute myocardial infarction; CHD, coronary heart disease; CVD, cardiovascular Disease; ECG, electrocardiogram; EPOCH, Evidence for Cardiovascular Prevention From Observational Cohorts; JALS, Japan Atherosclerosis Longitudinal Study.

^a^Values are expressed as median and interquartile range.

^b^Values indicate the number and proportion of participants in risk groups.

### Hisayama score

The Hisayama score demonstrates a similar upward trajectory in the 10-year risk of ASCVD (Table 1). In 2000, the absolute risk of ASCVD outcomes in 10 years was estimated at 2.56%, rising to 3.92% by 2019. Between 2000 and 2015, the estimated ASCVD risk increased sharply, reaching 4.52%, before showing a slight decline in 2019.

### Suita scores

The Suita score revealed a population-level shift toward higher cardiovascular risk over time (Table 1). From 2000 to 2019, the proportion of individuals in the lowest risk category (<1%) slightly decreased from 2.5% to 1.9%, while the largest group (2% risk) declined from 52.9% to 40.0%. In contrast, moderate-risk categories showed an upward trend: the 6% group increased from 16.0% to 18.7%, and the 9% group from 15.3% to 17.8%. Higher risk categories (14% and 25%) fluctuated slightly but remained a small increment. Including ECG in the Suita score did not drastically alter overall patterns compared with the Suita score without ECG. These trends suggest a gradual shift in cardiovascular risk distribution toward higher categories over the two decades, highlighting the growing need for population-level preventive interventions.

### JALS scores

Across all categories, JALS scores showed a gradual increase over time, regardless of AF status or disease type (stroke, AMI, composite outcomes of stroke and AMI, and CVD) (Table 1). For example, in stroke without AF, the score increased from 0.44% to 0.71%. AMI alone consistently exhibited low values throughout the observation period, from 0.10% to 0.15%, with minimal fluctuation. In contrast, composite outcomes and CVD categories started with relatively lower values but demonstrated gradual increases over time.

## DISCUSSION

We developed an R package, Jcvrisk, that allows simultaneous calculation of multiple CVD risk scores recommended in the JCS guideline. Using longitudinal data from the population-based cohort study, we assessed changes in CVD risk over the past 20 years.

All scores consistently indicated an upward trend in 10-year CVD risk. This trend may reflect changes in risk factors, such as hypertension, dyslipidemia, and T2DM, as well as shifts in lifestyle habits (eTable 1). On the other hand, improvements, such as a decrease in smoking rates and an increase in HDL-C, were observed; however, the proportion of individuals in the moderate-risk category or higher has increased overall, highlighting the growing importance of CVD prevention among the local population.

In brief, we compared CVD risk scores in our showcase with that reported in the original papers. Regarding EPOCH scores,^7^ a 10-year risk of death by CHD was slightly higher than stroke in the showcase even in same-age strata. This could result in differences in living environment and occupations between both populations. The Hisayama score (10-year risk of ASCVD incidence) in this showcase was normally distributed and almost the same as reported in the original paper, with mean values around 3–4%.^8^ Estimated 10-year absolute CVD risks in the top decile of Suita score (25%) seem to be little higher compared with those of the original study (less than 20%).^9^ All scores from the JALS showed relatively lower absolute risks for all outcomes in this showcase, but it is impossible to compare results in the original paper, in which the authors performed neither internal nor external validations. Given that the PREVENT score incorporates kidney function into risk calculation, a future CVD risk score in Japanese adults should consider including estimated glomerular filtration rate.^4^^,^^15^ Furthermore, genetic risk score (ie, polygenic risk score) is a must-see topic in terms of better risk stratification at a population level, even with a modest improvement in clinical settings.^16^^,^^17^ An updated function will be introduced for SCORE2 Asia-Pacific algorithms, which estimate a 10-year CVD event risk for healthy adults without prior CVD and T2DM.^18^

Given R is one of many statistical software programs used in medical research, we expect with future collaboration to expand to other software, including Python, SAS, and Stata. Another consideration could be interpretation of “risk” score. All scores in this package tell “XX% of incidence/mortality in the designated years (5 or 10 years)”. It may not be straightforward for both patients and medical staff. The future risk score should be more interpretable with considerations of preventive measures.

In conclusion, the Jcvrisk package is a useful tool for helping make a social treatment at population levels and accelerating cardiovascular epidemiology in Japan.

## SUPPLEMENTARY MATERIAL

eMaterial 1. Supplemental methods

eTable 1. Trends in risk factors of cardiovascular disease over a 20-year period
