# Supplementary material for: Jcvrisk: An R Package for Population-level Estimation of Cardiovascular Risk Scores in Japanese Adults
Source: J Epidemiol. 2026 Apr 5;36(4):148–52. doi: 10.2188/jea.JE20250292 (PMC12975768; doi:10.2188/jea.JE20250292)
Supplement: Supplementary file 1 [file je-36-148-s001.pdf]

eMaterial 1. Supplemental methods

Method 1: Calculation process for EPOCH-JAPAN risk score

**Risk Factors**	**CHD**	**Stroke**	**CVD**
Ln (age)	β_1 = 61.19918	β_1 = 37.40606	β_1 =45.54988
Men vs. Women	β_2 = 0.65869	-	β_2 = 0.30093
Current vs non-smoking	β_3 = 15.36389	β_3 = 8.24292	β_3 = 10.64932
Prevalent DM (+) vs. (-)	β_4 = 0.56252	β_4 = 0.45679	β_4 = 0.49413
Proteinuria (+) vs. (trace/-)	β_5 = 0.58243	β_5 = 0.63621	β_5 = 0.62120
Ln (SBP)	β_6 = 46.36999	β_6 = 26.39953	β_6 =33.30729
Ln (TC/HDL-C)	β_7 = 0.35931	-	-
Ln (age)× Ln (SBP)	β_8 = -10.61448	β_8 = -5.92290	β_8 =-7.54461
Ln (age)× Current smoking	β_9 = -3.51974	β_9 = -1.84505	β_9 = -2.41998
$So(t)$: baseline mortality	0.9981	0.9961	0.9942

10-year Absolute Risk = $\mathbf{1 - So(t)^{exp\ (\Sigma\beta x - \Sigma\beta\bar{x})}}$

$\boldsymbol{\Sigma\ \beta\bar{x}}$**:** Indicator of population characteristics.

Same values in the original paper were used in this package (CHD: 265.45, Stroke: 163.44, CVD: 198.25).

$\boldsymbol{\Sigma\ \beta x}$: Indicator of individual status for risk factor.

$\boldsymbol{\Sigma\ \beta x}$ = β_1 ×Ln(age) + β_2 × (male=1, female=0)

+ β_3 ×(DM=1, non-DM = 0) + β_4 × (Proteinuria (+) = 1, (-) = 0)

+ β_5 ×Ln(SBP) + β_6 × Ln([TC/HDL-C])

+ β_7 ×(Ln[age]×Ln[SBP])

+ β_8 × Ln(age)×(Current smoker = 1, non-smoker =0)

Calculation example of CHD mortality risk (Same example in the original paper)

Age	: 70
Sex	: female
Smoking	: non-smoker
Proteinuria	: (-)
DM	: (+)
SBP	: 135 mmHg
TC	: 185 mg/dL
HDL-C	: 58mg/dL

$\boldsymbol{\Sigma\ \beta x}$: = 61.19918×Ln(70) + 0.65869×0 + 15.36389×0 + 0.56252×1 + 0.58243×0 + 46.36999×Ln(135) + 0.35931×Ln(185/58)-10.61448×Ln(70)×Ln(135) -3.51974×Ln(70)×0=267.24

Absolute Risk of 10-year CHD mortality = $1-0.9981^{exp(267.24-265.45)}$ = 1.13%

Method 2: Calculation process for Hisayama risk score

Risk Factors	Score
Men	7
SBP < 120 mmHg	0
120-129 mmHg	1
130-139 mmHg	2
140-159 mmHg	3
160 mmHg -	4
HDL-C 60 mg/dL -	0
40-59 mg-dL	1
< 40 mg/dL	2
LDL-C <120 mg/dL	0
120-139 mg/dL	1
140-159 mg/dL	2
160 mg/dL -	3
No regular exercise	2
Diabetes	3
Proteinuria	4
Current smoker	2

10-year Absolute Risk = $1 - 0.9696^{exp\{(Total\ Score\ +\ Points\ for\ age)\ \times\ 0.144 - 2.4767\}}$

Total Score: Calculated based on each risk factor.

Points for Age: Points are assigned based on an individual's age (40-49:0, 50-59:5, 60-69:11,70-79:16, over 80:20)

Calculation example of 10-year ASCVD incidence risk

Age : 70

Sex : female

Smoking : non-smoker

Proteinuria : (-)

DM : (+)

Regular exercise : (-)

SBP : 135 mmHg

LDL-C : 145 mg/dL

HDL-C : 58mg/dL

Total Score : 0 + 0 + 0 + 3 + 0 + 2 + 2 + 1 + = 8

Point for Age : 16

Absolute Risk of 10-year ASCVD incidence = $1 - 0.9696^{exp\{(8\ +\ 16)\ \times\ 0.144 - 2.4767\}}$ = 7.89%

Method 3: Calculation process for Suita risk score

Risk Factors	With ECG	Without ECG
Men	4	4
Age 30-39	0	0
40-49	8	8
50-59	14	14
60-64	18	18
65-69	22	22
70-74	25	26
75-79	28	29
SBP < 120 mmHg and DBP < 80 mmHg	-4	-4
SBP = 120-139 mmHg and DBP = 80-89 mmHg	0	0
SBP = 140-159 mmHg and DBP = 90-99 mmHg	3	3
SBP ≥ 160 mmHg or DBP ≥ 100 mmHg or in medication	6	6
Non-HDL-C < 170mg/dL and LDL-C < 140 mg/dL	0	0
Non-HDL-C ≥ 170mg/dL or LDL-C ≥ 140 mg/dL	2	2
HDL-C < 40 mg/dL	0	0
40-59 mg/dL	-2	-2
≥ 60 mg/dL	-4	-4
Smoking	4	4
DM	6	7
Urinary protein 1+ or more	2	2
Atrial fibrillation	8	-
Left ventricular hypertrophy	5	-

10-year absolute risk classified by total score

Total Score	With ECG	Without ECG
0 or less	Less than 1%	Less than 1%
1-20	2%	2%
26-30	6%	6%
21-25	9%	9%
31-35	15%	14%
36 or more	26%	25%

Calculation Example of 10-year CVD incidence risk in without ECG model

Age : 70

Sex : female

Smoking : non-smoker

Proteinuria : (-)

DM : (+)

SBP : 135 mmHg

TC : 285 mg/dL

LDL-C : 140 mg/dL

HDL-C : 58 mg/dL

Total Score : 25 + 0 + 0 + 0 + 7 - 2 + 2 = 32

Absolute Risk of 10-year CVD incidence = 14%

Method 4: Calculation process for JALS risk score

Risk Factors			Stroke		AMI		Stroke + AMI		CVD	
			AF(-)	AF(+)	AF(-)	AF(+)	AF(-)	AF(+)	AF(-)	AF(+)
Atrial fibrillation		No	-	0	-	0	-	0	-	0
		Yes	-	21	-	12	-	20	-	18
BMI		<18.5	-	-	-	-	-	-	6	7
(kg/m^2)		18.5-25.0	-	-	-	-	-	-	0	0
		25≤	-	-	-	-	-	-	-1	-2
HDL cholesterol		<40	-	-	11	11	4	4	3	3
(mg/dL)		40-59	-	-	7	7	2	2	-1	-1
		60≤	-	-	0	0	0	0	0	0
Blood pressure (SBP/DBP)	No antihypertensive	< 120 and < 80	0	0	0	0	0	0	0	0
(mmHg)	medication	120-129 and/or < 80	8	8	4	4	7	7	0	0
		130-139 and/or 80-89	7	7	4	4	7	7	3	3
		140-159 and/or 90-99	14	14	7	7	13	14	4	4
		160-179 and/or 100-109	21	21	9	9	19	19	10	11
		≥180 and/or ≥ 110	27	27	17	17	26	26	13	13
	medication	< 120 and < 80	12	12	2	2	11	10	7	6
		120-129 and/or < 80	13	13	15	15	14	13	7	7
		130-139 and/or 80-89	14	14	16	16	14	14	5	5
		140-159 and/or 90-99	18	18	11	11	17	17	6	6
		160-179 and/or 100-109	19	19	6	6	17	18	8	9
		≥ 180 and/or ≥ 110	19	19	9	9	18	18	5	5
Age		40-49	0	0	0	0	0	0	0	0
(years old)		50-59	4	4	11	11	6	5	6	6
		60-69	13	13	14	13	13	13	16	16
		70-79	24	23	21	21	23	23	35	35
		80 ≤	31	31	28	27	31	30	56	55
eGFR		<45	-	-	-	3	-	-	11	11
($mL/min/1.73m^2$)		45-60	-	-	-	3	-	-	2	2
		60-90	-	-	-	0	-	-	0	0
		90≤	-	-	-	0	-	-	1	1
Non- HDL cholesterol		<130	-	-	0	0	-	-	-	-
(mg/dl)		130-149	-	-	10	10	-	-	-	-
		150-169	-	-	13	13	-	-	-	-
		170≤	-	-	17	17	-	-	-	-
Diabetes Mellitus		No	0	0	0	0	0	0	0	0
		Yes	6	6	9	9	6	6	4	4
Sex		Men	5	4	17	17	6	5	6	6
		Women	0	0	0	0	0	0	0	0
Current		No	0	0	0	0	0	0	0	0
smoking		Yes	7	7	10	11	7	8	9	9

5-year absolute risk classified by total score across age categories.

Score[a]	40-49	50-59	60-69	70-79	80-89
			Age categories		
			Stroke Model (AF is included)		
**0**	0.11%	0.15%	0.27%	0.55%	0.93%
**5**	0.16%	0.21%	0.38%	0.78%	1.31%
**10**	0.22%	0.29%	0.54%	1.10%	1.85%
**15**	0.32%	0.42%	0.76%	1.55%	2.60%
**20**	0.45%	0.59%	1.07%	2.18%	3.66%
**25**	0.63%	0.83%	1.51%	**3.08%**	5.14%
**30**	0.89%	1.17%	2.13%	4.32%	7.19%
**35**	1.26%	1.65%	3.01%	6.06%	10.02%
**40**	1.78%	2.33%	4.22%	8.46%	13.86%
**45**	2.50%	3.27%	5.92%	11.75%	19.03%
**50**	3.52%	4.60%	8.27%	16.20%	25.81%
**55**	4.94%	6.44%	11.49%	22.11%	34.43%
**60**	6.92%	8.98%	15.85%	29.77%	44.95%

[a]Score means subtract values total score from age score (score 0 means having no risk factors excluding age). Extremely high or low scores (e.g. less than 0 or greater than 60) are treated as equivalent to the upper and lower limits. Therefore, the 5-year absolute risk for a score of 65 is the same as for a score of 60.

Calculation Example of 5-year Stroke risk in with AF model

Age : 75

Sex : man

SBP : 160–179 mmHg

DBP : 100–109 mmHg

Hypertension medication : no medication

DM : (-)

Smoking : non-smoker

Total Score : 23 + 4 + 21 + 0 + 0 = 48 (25 after subtracting age score of 23)

Absolute Risk of 5-year stroke incidence = 3.08%

(The corresponding value in the above table is 25 points for the 70-79 age category)

eTable 1. Trends in risk factors of cardiovascular disease over a 20-year period

	2000 (N=437)	2005 (N=343)	2010 (N=331)	2015 (N=329)	2019 (N=315)
Age	59 (10)	61 (10)	63 (10)	63 (10)	63 (10)
Women	270 (61.8%)	226 (65.9%)	204 (61.6%)	180 (54.7%)	190 (60.3%)
SBP, mm Hg	126.1 (17.4)	134.3 (19.4)	130.9 (17.8)	132.0 (20.8)	138.2 (20.6)
DBP, mm Hg	78.9 (9.9)	80.9 (10.7)	77.5 (10.4)	76.6 (13.8)	78.3 (12.3)
BMI, kg/m^2	23.7 (3.0)	23.5 (3.1)	23.3 (3.1)	23.8 (3.3)	23.7 (3.6)
Triglyceride, mg/dL[a]	92.0 [71.0, 132.0]	90.0 [66.5, 127.5]	87.0 [66.0, 116.0]	88.0 [68.0, 127.0]	85.0 [58.0, 114.5]
Total cholesterol, mg/dL	207.1 (32.9)	211.2 (32.0)	201.7 (29.1)	212.4 (33.7)	210.4 (32.8)
HDL-C, mg/dL	53.5 (16.2)	59.9 (13.9)	56.0 (12.6)	58.8 (13.4)	63.5 (15.1)
LDL-C, mg/dL	132.1 (32.9)	130.9 (29.6)	124.8 (27.5)	133.0 (30.0)	127.7 (30.3)
eGFR, mL/min/1.73m^2	85.5 (20.2)	79.3 (16.0)	66.2 (13.4)	71.7 (13.6)	71.0 (13.6)
T2DM	33 (7.6%)	18 (5.2%)	27 (8.2%)	45 (13.7%)	31 (9.8%)
Proteinuria	19 (4.3%)	30 (8.7%)	42 (12.7%)	52 (15.8%)	20 (6.3%)
Regular exercise	97 (22.2%)	103 (30.0%)	63 (19.0%)	64 (19.5%)	58 (18.4%)
Current smoker	105 (24.0%)	50 (14.6%)	37 (11.2%)	50 (15.2%)	49 (15.6%)
Atrial fibrillation	5 (1.1%)	1 (0.3%)	1 (0.3%)	1 (0.3%)	0 (0.0%)
Left ventricular hypertrophy	2 (0.5%)	3 (0.9%)	8 (2.4%)	2 (0.6%)	1 (0.3%)

BMI, body mass index; DBP: diastolic blood pressure; eGFR, estimated glomerular filtration rate; HDL-C, high-density lipoprotein cholesterol; LDL-C, low-density lipoprotein cholesterol; SBP, systolic blood pressure; T2DM, type 2 diabetes mellitus.

[a] Median [Interquartile range]
